# Human *in vitro* assay for irreversible electroporation cardiac ablation

**DOI:** 10.3389/fphys.2022.1064168

**Published:** 2023-01-09

**Authors:** Maura Casciola, Tromondae K. Feaster, Michael J. Caiola, Devin Keck, Ksenia Blinova

**Affiliations:** Office of Science and Engineering Laboratories, Center for Devices and Radiological Health, United States Food and Drug Administration, Silver Spring, MD, United States

**Keywords:** induced pluripotent stem cell-derived cardiomyocyte (hiPSC-CM), irreversible electroporation (IRE), electroporation, cardiomyocytes, pulsed field ablation PFA, *in vitro* assay

## Abstract

**Introduction:** Pulsed electric field (PEF) cardiac ablation has been recently proposed as a technique to treat drug resistant atrial fibrillation by inducing cell death through irreversible electroporation (IRE). Improper PEF dosing can result in thermal damage or reversible electroporation. The lack of comprehensive and systematic studies to select PEF parameters for safe and effective IRE cardiac treatments hinders device development and regulatory decision-making. Human induced pluripotent stem cell-derived cardiomyocytes (hiPSC-CMs) have been proposed as an alternative to animal models in the evaluation of cardiac electrophysiology safety.

**Methods:** We developed a novel high-throughput *in vitro* assay to quantify the electric field threshold (EFT) for electroporation (acute effect) and cell death (long-term effect) in hiPSC-CMs. Monolayers of hiPSC-CMs were cultured in high-throughput format and exposed to clinically relevant biphasic PEF treatments. Electroporation and cell death areas were identified using fluorescent probes and confocal microscopy; electroporation and cell death EFTs were quantified by comparison of fluorescent images with electric field numerical simulations.

**Results:** Study results confirmed that PEF induces electroporation and cell death in hiPSC-CMs, dependent on the number of pulses and the amplitude, duration, and repetition frequency. In addition, PEF-induced temperature increase, absorbed dose, and total treatment time for each PEF parameter combination are reported.

**Discussion:** Upon verification of the translatability of the *in vitro* results presented here to *in vivo* models, this novel hiPSC-CM-based assay could be used as an alternative to animal or human studies and can assist in early nonclinical device development, as well as inform regulatory decision-making for cardiac ablation medical devices.

## 1 Introduction

Cardiac catheter ablation is a standard electrophysiological procedure for treatment of atrial fibrillation (AF) in patients who are non-responsive to medications (Wilber et al., 2010; Khan et al., 2014; Santangeli et al., 2014; Al-Khatib et al., 2020). AF can lead to serious complications, including blood clots, stroke, and heart failure. The number of people in the United States affected by AF is projected to increase from 2.3 million to more than 10 million by the year 2050 (Chen and Shen, 2007). Annually, approximately 75,000 people in the United States are estimated to undergo cardiac ablation (Mansour et al., 2017).

Current state-of-the-art ablation modalities, including radiofrequency (RF) ablation and cryoablation, rely on tissue heating or cooling to destroy the aberrant arrhythmic cardiac substrate (Habibi et al., 2021; Calvert et al., 2022). While these modalities are relatively non-invasive and are considered safe and effective, they have significant limitations. These include high AF recurrence rates (Balk et al., 2010; Sultan et al., 2017), prolonged procedure times, and rare but serious complications related to the damage of off-target neighboring structures, such as pulmonary vein (PV) stenosis, phrenic nerve paralysis, and fatal esophageal fistula (Bertaglia et al., 2007; Spragg et al., 2008; Kearney et al., 2014; Calkins et al., 2017; Steinbeck et al., 2018; Samuel et al., 2019).

Pulsed electric field (PEF) cardiac ablation, also known as pulsed field ablation (PFA), has been proposed as an alternative to thermal ablation for its use of irreversible electroporation (IRE) to achieve the therapeutic effect (Reddy et al., 2018; Maor et al., 2019; Sugrue et al., 2019; De Potter et al., 2021; Verma et al., 2021; Gunawardene et al., 2022). When PEF waveform parameters such as pulse amplitude, duration, number, and repetition frequency are properly selected, PEF results in non-thermal cell permeabilization, also known as electroporation (Davalos et al., 2005; Miklavcic and Davalos, 2015; Aycock and Davalos, 2019). Cell electroporation produces changes in ionic concentration gradients, and thus in the cell homeostasis, that can reach sufficient duration or intensity to generate irreversible structural changes, leading to either immediate necrosis or programmed cell death processes (Batista Napotnik et al., 2021). Taken together, PEF cardiac ablation, by avoiding thermal effects, has the potential to improve patient safety and enables the treatment of areas where procedures would be challenging due to the risk of off-target damages associated with thermal ablation approaches.

PEF preclinical studies on animals have been performed on epicardium, PVs, endocardium, coronary vessels, cardiac ganglia, and surrounding structures, such as the phrenic nerve and esophagus (Lavee et al., 2007; Hong et al., 2009; Zager et al., 2016; Stewart et al., 2019; Caluori et al., 2020; Stewart et al., 2021). Recently, the first published report of acute clinical experience of PEF cardiac ablation on humans in the United States suggested that PEF-based PV ablation is clinically feasible (Reddy et al., 2018). Nevertheless, several factors need to be addressed to optimize PEF cardiac ablation and enable its widespread use (Verma et al., 2021). Specifically, PEF parameters and electrode configurations should be carefully selected to optimize therapeutic effects and minimize potential risks, such as undesired muscular contraction and thermal heating (Arena et al., 2011). For example, studies have demonstrated that short biphasic pulses (i.e., microsecond range) can reduce electrolytic contamination (Kotnik et al., 2001), neuromuscular stimulation, and pain (Fusco et al., 2021), while maintaining electroporation efficacy; thus, this pulse shape is commonly used in the clinic (Reddy et al., 2018; Reddy et al., 2020a; Reddy et al., 2020b; Kawamura et al., 2021a; Kawamura et al., 2021b; Nakatani et al., 2021; Reddy et al., 2021; Verma et al., 2022). However, optimal treatment protocols are yet to be determined.

Preclinical studies aimed at quantifying the electric field threshold (EFT) for cardiac electroporation used various PEF parameters and animal models (Xie et al., 2015; Xie and Zemlin, 2016; Semenov et al., 2018; Azarov et al., 2019; Neuber et al., 2019; Heller et al., 2021). These publications suggest that EFTs for electroporation are treatment dependent. However, the lack of comprehensive and systematic investigations to assess EFTs of cardiac tissues for a range of PEF parameters, together with the lack of human *in vitro* models to evaluate PEF cardiac ablation device safety and effectiveness, delays regulatory decisions and places a significant burden on animal models (Harris et al., 2013). A recent study in which AC16 cells were exposed to a limited range of PEF treatments showed how human cardiomyocytes in a monolayer culture could be used to determine the relationship between ablation threshold and PEF parameters (Baena-Montes et al., 2022). While the study supports the feasibility of *in vitro* assessment of PEF cardiac ablation, AC16 expression patterns of mRNA profile and cardiomyocyte markers showed low similarity to primary cells regardless of the differentiation method adopted (Gulyas-Onodi et al., 2022).

On the contrary, patient-derived human induced pluripotent stem cells-derived cardiomyocytes (hiPSC-CMs) show molecular, mechanical, electrophysiological, metabolic, and ultra-structural properties similar to primary cells (Yang et al., 2014; Ribeiro et al., 2015). In fact, recent results demonstrated that hiPSC-CMs are a more appropriate model for *in vitro* studies than cell lines due to their higher similarity to adult cardiac tissue (Onodi et al., 2022). Both 2D and 3D formats have been increasingly used as *in vitro* platforms for preclinical drug screening and development. These approach has been validated in multi-site studies (Blinova et al., 2018) including chronic studies (Narkar et al., 2022a), and the best practice recommendations for use of these methods in drug safety assessment has been published (Gintant et al., 2020). More recently, hiPSC-CMs have been proposed as a valid alternative to animal models in the evaluation of cardiac electrophysiology medical devices (Casciola et al., 2020; Feaster et al., 2021; Casciola et al., 2022; Feaster et al., 2022; Narkar et al., 2022b). Here, we establish a novel, species-relevant, standard laboratory protocol to evaluate and optimize PEF cardiac ablation parameters using hiPSC-CMs in a high-throughput monolayer format.

This work is part of a larger effort to improve regulatory decision-making and support safety and effectiveness studies of cardiac electrophysiology medical devices. Here, we demonstrate that hiPSC-CMs respond to changes in pulse duration, number, amplitude, and repetition frequencies by acute, i.e., reversible, and long term, i.e., irreversible, electroporation. We systematically varied one parameter at a time to quantify electroporation and cell death EFTs, as well as possible thermal increase. This unique preclinical *in vitro* assay provides a foundation for PEF cardiac ablation treatment planning and optimization, with the potential to accelerate device development and the regulatory review process.

## 2 Materials and methods

### 2.1 Cell culture and maintenance

HiPSC-CMs, iCell Cardiomyocytes^2^ catalog # 01434 (Fujifilm Cellular Dynamics, Inc., Madison, WI), were handled according to the manufacturer’s instructions (fujifilmcdi, 2022). HiPSC‐CMs used in this study were derived from the hiPSC line, which was reprogrammed from fibroblast donor tissue isolated from an apparently healthy normal Caucasian female <18 years old (Ma et al., 2011; Feaster et al., 2021). Briefly, hiPSC-CMs, in a concentration of 110,000–115,000 cells per well, were plated on 96-well Nanofiber plates, catalog # 9602 (Nanofiber Solutions, Dublin, OH), coated with Matrigel substrate, catalog # 356230 (Corning Inc., Somerville, MA), in a 1:60 DMEM dilution, catalog # 30-2006 (ATCC, Manassas, VA). Cells were maintained using iCell Cardiomyocytes Maintenance Medium, catalog #M1003 (Fujifilm Cellular Dynamics, Inc.). Spontaneously beating, 100% confluent hiPSC-CM monolayers were used for PEF treatment testing on days 7–14 after plating. Hoechst-33342 (Ho) (2.25 µM), catalog #H3570 (Invitrogen, ThermoFisher Scientific, Waltham, MA) dye, labeling the nuclei of all cells, was used to assess monolayer confluency and integrity before pulsing (see Supplementary Figure S1). Two solutions were used during cell staining, PEF treatment, and imaging: iCell Cardiomyocytes Serum-Free Medium (iCM-SF), catalog #M1038, (Fujifilm Cellular Dynamics, Inc.), or modified Tyrode’s solution containing (in mM) 140 NaCl, 5 KCl, 2 CaCl_2_, 2 MgCl_2_, 5 HEPES, 10 Glucose, pH 7.4 with NaOH and electric conductivity of 1.8 S/m at room temperature, and 2.3 S/m at 37°C. PH and conductivity were measured with an S47 SevenMulti dual meter pH/conductivity (Mettler Toledo, Columbus, OH).

### 2.2 PEF treatments delivery

A pair of custom stainless-steel needle electrodes (.61 mm diameter, 1.7 mm distance center to center) were connected to an electric pulse generator, model FPG 1B50-1UL10 (FID GmbH, Germany) that was controlled with a digital delay generator, model 577-4C (Berkeley Nucleonics Corporation, San Rafael, CA). As in previous reports (Gudvangen et al., 2022), we used a 3D printer, model Anet A8 (Shenzhen Anet Technology Co., China), as an automated robotic arm for accurate positioning of the electrodes orthogonally to the cell monolayer; the tip of the electrodes was in contact with the bottom of the well. An electrode holder, connected to the 3D printer, was equipped with a spring system to minimize the pressure of the electrodes on the bottom of the plate. The 3D printer was programmed to move the electrodes to the center of each well with a 15–50 s delay, depending on the PEF parameter selection. The Anet A8 stage was heated to 50°C to adjust the pretreatment temperature of the Tyrode solution to 37.5°C ± 1.0°C. A multi-well plate holder (Olympus, Center Valley, PA) was secured, i.e., glued, to the 3D printer stage to avoid relative movements between electrodes and the multi-well plate. Pulse shape and amplitude were monitored with an oscilloscope (Tektronix, Beaverton, OR) connected to a 1:100 voltage probe, model P2501 (Owon Technology Inc., China) and an electric current probe, model TEK TCP 2020 (Tektronix).

**FIGURE 1:**
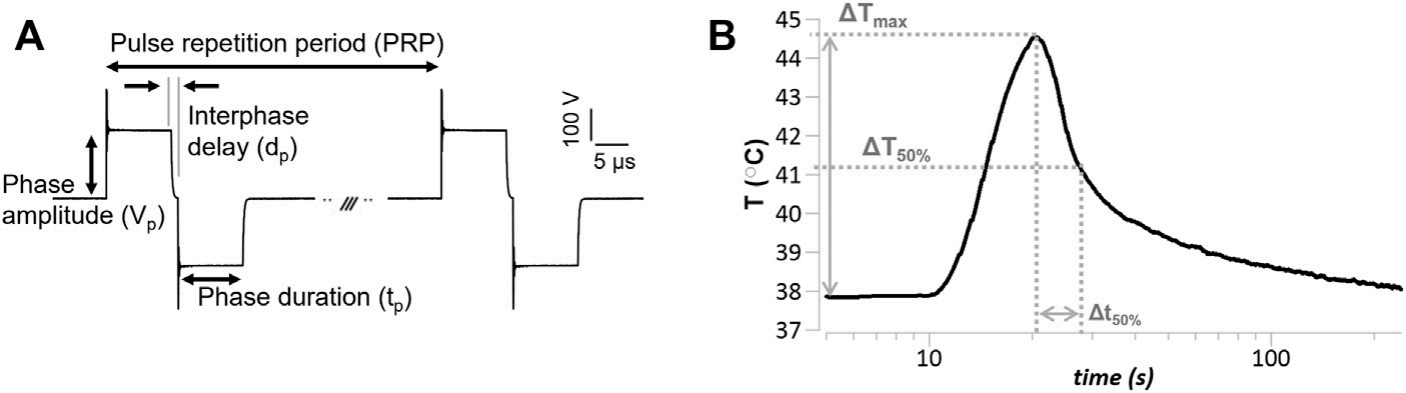
Pulsed electric field (PEF) pulse parameters and temperature endpoints **(A)** Representative voltage waveform (V_p_ = 236 V, t_p_ = 10 µs, d_p_ = 1 µs, PRP = .1 s), and relevant PEF parameters definition. The pulse repetition period is the inverse of the pulse repetition frequency reported in the text. The voltage amplitude overshoot was 20%–60% of the phase amplitude. **(B)** Representative temperature measure in response to a PEF treatment (V_p_ = 236 V, t_p_ = 10 µs, *p*_#_ = 100, PRF = 10 Hz) delivered at approximately 10 s from the beginning of the recoding; depicted are relevant temperature endpoints, i.e., maximum PEF-induced temperature increase (ΔT_max_), and time to 50% temperature recovery (Δt_50%_).

A schematic of the experimental setup is reported in Supplementary Figure S2. The biphasic electric pulses were characterized by the following parameters: pulse repetition frequency (PRF), defined as the inverse of the pulse repetition period (PRP); number of biphasic pulses (*p*_#_) delivered in a single train; phase amplitude (V_p_); phase duration (t_p_); and interphase delay (d_p_) (Figure 1A). For each combination of PEF parameters, V_p_ was gradually increased until a clear measurable electroporation region was produced, while avoiding higher values of V_p_ that caused cell detachment and monolayer dissociation (see an example in Supplementary Figure S3). The interphase delay was maintained for all the PEF combinations at 1 µs.

### 2.3 PEF-induced temperature measurements

Thermal changes due to PEF treatments were measured using a non-metallic optic STB probe, catalog # L-00-14500-01 (Advanced Energy Industries, Denver, CO) with a response time of .25 s, sampling rate of .02 s, and diameter of .5 mm. The STB probe was positioned adjacent and parallel to one of the electrodes (Supplementary Figure S2). To eliminate the influence of the STB probe on the PEF treatments, temperature measurements were carried out in cell-free plates separately from the experiments used for performing the PEF treatment analysis, as previously described (Arena et al., 2012). A representative temperature measure over time and the endpoints defined as maximum temperature increase (ΔT_max_), 50% temperature recovery (ΔT_50%_), and time-to-50%-recovery (Δt_50%_) are shown in Figure 1B.

### 2.4 Fluorescence imaging

**FIGURE 2:**
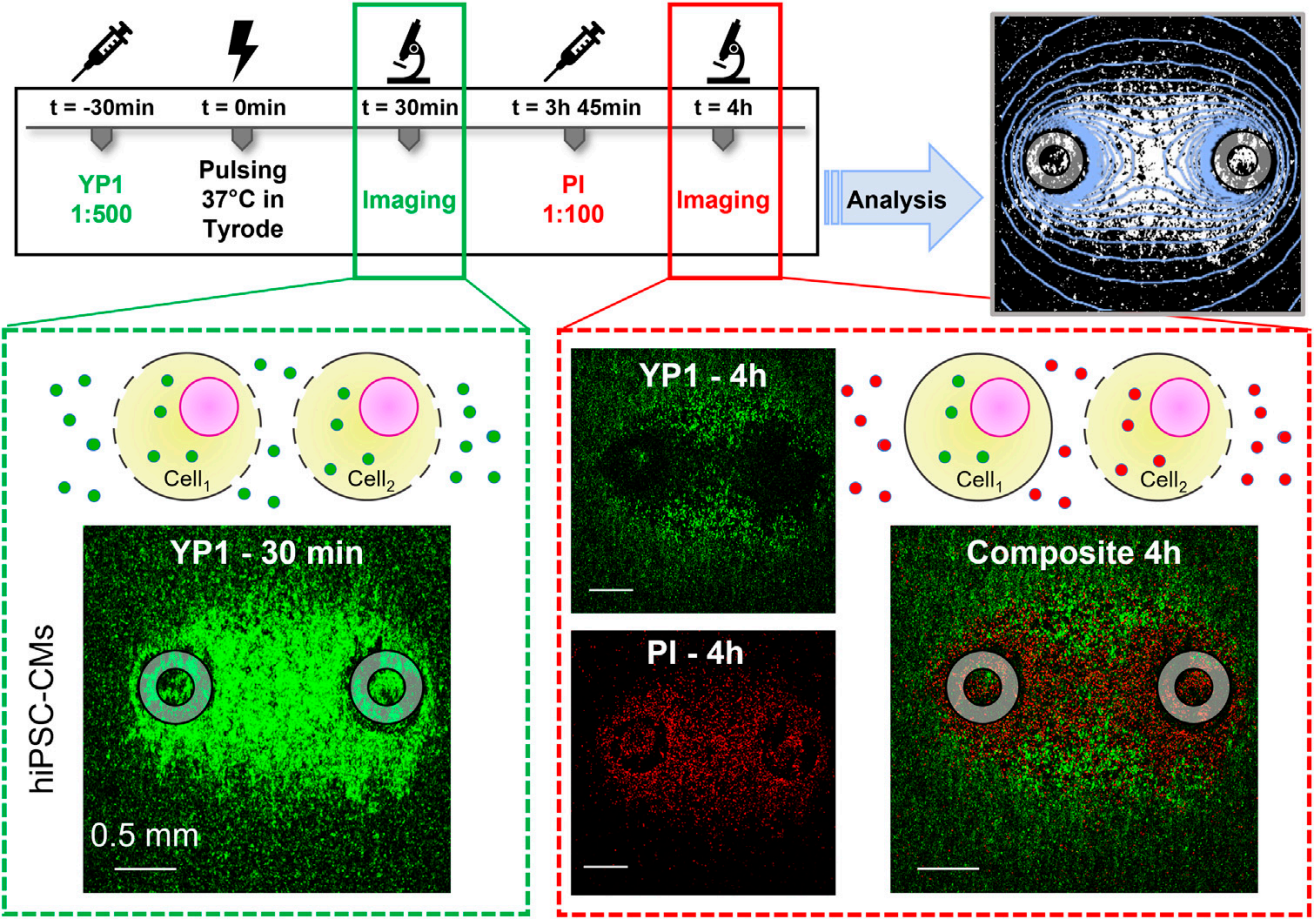
Human induced pluripotent stem cell-derived cardiomyocyte (HiPSC-CM) staining, electroporation, imaging, and analysis timeline. YP1 was added to the modified Tyrode solution 30 min prior to PFE treatments. YP1 images were acquired 30 min after PFE treatment to evaluate the full extent of cell electroporation. Immediately after imaging, cells were moved to a 37°C 5% CO_2_ incubator in iCM-SF. During this time, the plasma membrane of cells reversibly electroporated resealed. Fifteen minutes prior the 4 h imaging, iCM-SF was substituted by modified Tyrode solution containing PI staining permanently damaged cell membranes. Overlay of green and red channels showed a PI-stained area surrounding the electrodes and a peripheral outer YP1-stained area of the electroporated cells. The transition between these regions was gradual, with a resulting region where cells stained with YP1 and cells stained with PI were detected. Analysis was performed for YP1 and PI staining at the end of the experiments to compare the areas identified by fluorescence staining to the area identified by electric field isolines (light blue). Gray circles indicate the footprints of the electrodes positioned orthogonally to the cell monolayer.

PEF-treated hiPSC-CM monolayers were stained with two cell-impermeable fluorescent probes: YO-PRO-1 Iodide (YP1) (5 µM) and Propidium Iodide (PI) (15 µM), catalog numbers Y3603 and P3566, respectively, (Invitrogen, ThermoFisher Scientific), at different times (Figure 2). 15 min before experimental cells were washed with 200 µl per well of dulbecco’s phosphate-buffered saline DPBS, catalog number 14190-144 (Gibco, ThermoFisher Scientific). DPBS was immediately replaced with 100 µl per well of modified Tyrode solution containing YP1. Stained cells by YP1 were imaged using confocal microscopy 30 min after PEF treatment. After imaging, the Tyrode solution was changed to iCM-SF, 100 µl per well, and plates were returned to a 37°C, 5% CO_2_ cell culture incubator. To stain dead cells, iCM-SF was substituted with Tyrode containing PI 15 min prior imaging and imaged .5, 2, 4, 6, and 24 h after PEF treatment. The PI-stained area increased up to 2–4 h after PEF treatment, without further expansion at later time points (Supplementary Table S1).

For all the experiments described here, the 2–4 h timepoint was used to assess cell death areas. The laser scanning confocal microscope, model FluoView 3000 (Olympus) (Supplementary Figure S2) equipped with an environmental chamber (i.e., 37°C and 5% CO_2_), model OKO-H301-OLY-IX3-SVR (Okolab stl, Italy), was used to collect fluorescent images from multiple wells using a 4X, NA/0.16 dry objective. The following settings and conditions were chosen: resonant one-way scanning mode with four times the average line and sequential scan per line (.067 µs/pixel, 520.025 ms/frame); YP1 was excited with a 488 nm laser and the emission of the dye was detected in the 488–540 nm range (varied to maximize signal for each plate); PI emission was excited with a 561 nm laser and detected in the 570–670 nm range (all plates).

### 2.5 Analysis of electroporation and cell death areas

To quantify the electroporated area, YP1-stained regions were analyzed with ImageJ software (NIH, Bethesda, MD) (Schneider et al., 2012). Images were converted to an 8-bit binary format (background threshold 12% ± 1%). Stacks of binary images were used as input to the Analyze Particle function that identified and quantified the area of the YP1-stained regions. The electrodes’ imprint area was quantified from sham exposures, i.e., .94 ± .18 mm^2^, and subtracted from the electroporated area when needed.

PI-stained regions were analyzed using a custom MATLAB (MathWorks Inc., Natick, MA) code (Supplementary Appendix S1) that accounted for the gradual decrease in PI fluorescence at the border of the irreversibly electroporated region. Our code discretized each image in a matrix of 128 × 128 elements; for each element, the sum of red pixels with intensity above background (i.e., approximately 5%–15% of maximum intensity) was computed and normalized to 100%. The IRE area was obtained according to Eq. 1, multiplying the total number (N) of pixels (px) above a threshold value (*th*) by a conversion factor pixel^2^ to mm^2^ (*c*_*f*_).

$$IRE\;area=c_{f}\sum_{i=1}^{128x128}N_{i\left(px>th\right)}$$ (1)

In our analysis, to identify the margin of cell death area excluding regions where both YP1 and PI staining were present, *th* was set as 30% of the maximum density count (Supplementary Figure S4).

### 2.6 Numerical simulations and estimation of electroporation and cell death EFTs

The external edge of the electroporation and cell death areas correspond to the minimum electric field, i.e., threshold, necessary to induce the effect. In this study, electroporation and cell death EFTs were identified by comparing the areas identified by YP1 and PI staining, respectively, to the electric field distribution from numerical simulations (Arena et al., 2012; Neal et al., 2014; Aycock et al., 2021; Liu et al., 2021; Aycock et al., 2022). The finite element analysis software Comsol Multiphysics 5.6 (COMSOL Inc., Stockholm, Sweden) was used to solve in static conditions the applied electric field map in the cell monolayer plane during pulsing. A 3D geometry of our pulse delivery system was constructed with equivalent dimensions for the electrodes and well used in the experimental setup (Supplementary Figure S5).

Each treatment was performed in a single well of a 96-well plate with a thickness of 1 mm and a radius of 3.5 mm. Stainless-steel electrodes (4∙10^6^ S/m) were placed perpendicular to the bottom of the well and inserted in a 2.75 mm thick layer of water with conductivity 2.3 S/m mimicking the modified Tyrode solution at 37°C. The geometry was surrounded by a cube of air (15 mm^3^ × 15 mm^3^ × 15 mm^3^, 0 S/m). The “finer” mesh setting, resulting in 166,212 elements, was used, with 5,910 elements for the electrodes (element volume ratio .0419) and 27,357 elements for Tyrode solution (element volume ratio 7∙10^−3^). One electrode was set to an electric potential equal to the phase amplitude of the PEF treatments, while the other electrode was set to 0 V. Electric field contours at varying magnitudes were created for each treatment, and the surface area within each contour was integrated with steps of 1 V/cm. Similarly to (Aycock et al., 2022), the curve fitting tool in MATLAB was used to fit a two-term exponential function to the resulting area versus electric field data. Finally, measured electroporation and cell death areas were used as inputs to this function to compute respective EFTs.

To estimate the temperature increase in adiabatic conditions, the adiabatic heating (AH, °C) was derived from the absorbed dose (AD, mJ/g) calculated according to (Ibey et al., 2009). Briefly, the following relationships were used:

AH=AD0.24/1000 (2)

$$AD=\sigma \frac{E^{2}}{\rho }\cdot \tau \cdot 10^{6}$$ (3)

where *τ* = 2t_p_ is the pulse duration (s), E is the electric field in the solution (kV/cm) at the cell death threshold, *ρ* is the density of the solution (∼1 g/cm^3^), and *σ* is the conductivity of the solution (mS/cm).

### 2.7 Statistical analysis

Data are presented as mean ± standard error. Electroporation and cell death areas, as well as derived endpoints for a given set of PEF parameters were calculated as an average for *n* = 3–8 per group. In Figure 4, statistical analysis was performed using the Mann Whitney Test for unpaired data, significance was determined to be *p* < .05 before Bonferroni correction. Temperature measurements were calculated as an average for *n* = 3 per group.

## 3 Results

### 3.1 PEF treatments induce electroporation and cell death in HiPSC-CMs

**FIGURE 3:**
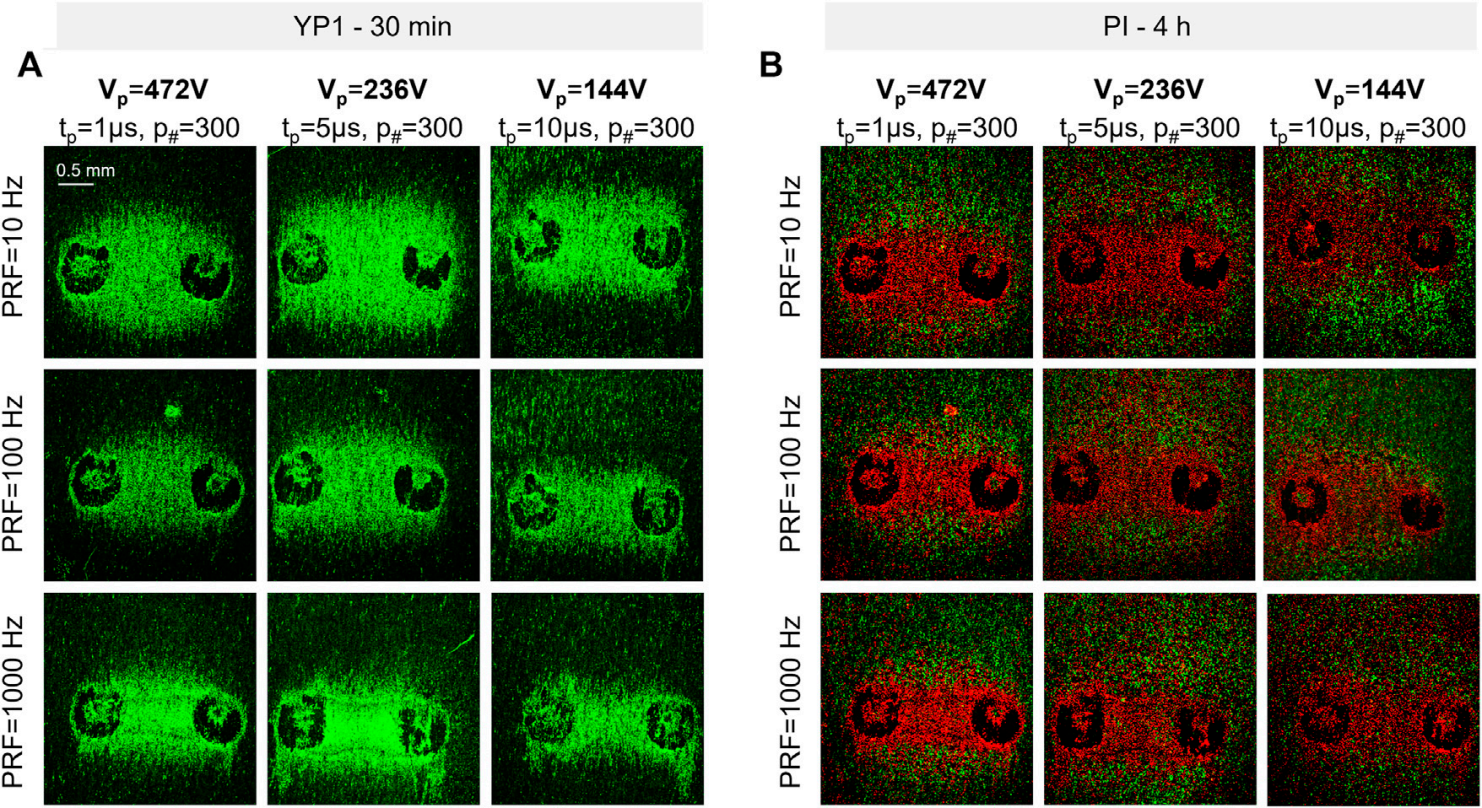
Representative fluorescent images of PEF-induced electroporation in hiPSC-CM monolayers **(A)** The green areas between the electrodes correspond to the location of electroporated hiPSC-CM monolayers 30 min after treatment. **(B)** The red area between the electrodes correspond to the location of dead hiPSC-CMs 4 h after treatment. The green staining at the edge of the electroporated area 4 h after treatment corresponds to the reversibly electroporated cells. The 488 laser intensity was different in A and B to highlight YP1-stained cells at 4 h, thus background fluorescence is more evident in **(B)**. Two black circles on each image represent the imprints of the electrodes.

We first evaluated the effects of PEF treatments (Figure 1A) in hiPSC-CMs. Robust electroporation and cell death of the hiPSC-CM monolayers was observed by fluorescence staining after PEF application (Figure 2). YP1 staining 30 min after treatment in response to trains of *p*_#_ = 300 delivered at PRF = 10, 100, and 1,000 Hz, V_p_ = 144, 236, and 472 V, and t_p_ = 1, 5, and 10 µs, resulted in a PEF parameter-dependent uptake (Figure 3A). In fact, the area of electroporation in hiPSC-CMs varied with changes in PEF parameters. The cell death areas indicated by delayed PI staining (Figure 3B) followed the same PEF parameter-dependent trend as areas indicated by YP1 (Figure 3A). However, cell death areas, labeled PI, were smaller than electroporated areas, labeled YP1, consistent with the expectation that cells located further from the electrodes, and therefore exposed to a lower electric field, would be more capable of repair (reversible electroporation). These results demonstrated that PEF treatments elicit detectable electroporation and cell death in an *in vitro* human cardiomyocyte model.

### 3.2 Effect of PEF parameters on cell electroporation and death in HiPSC-CMs

**FIGURE 4:**
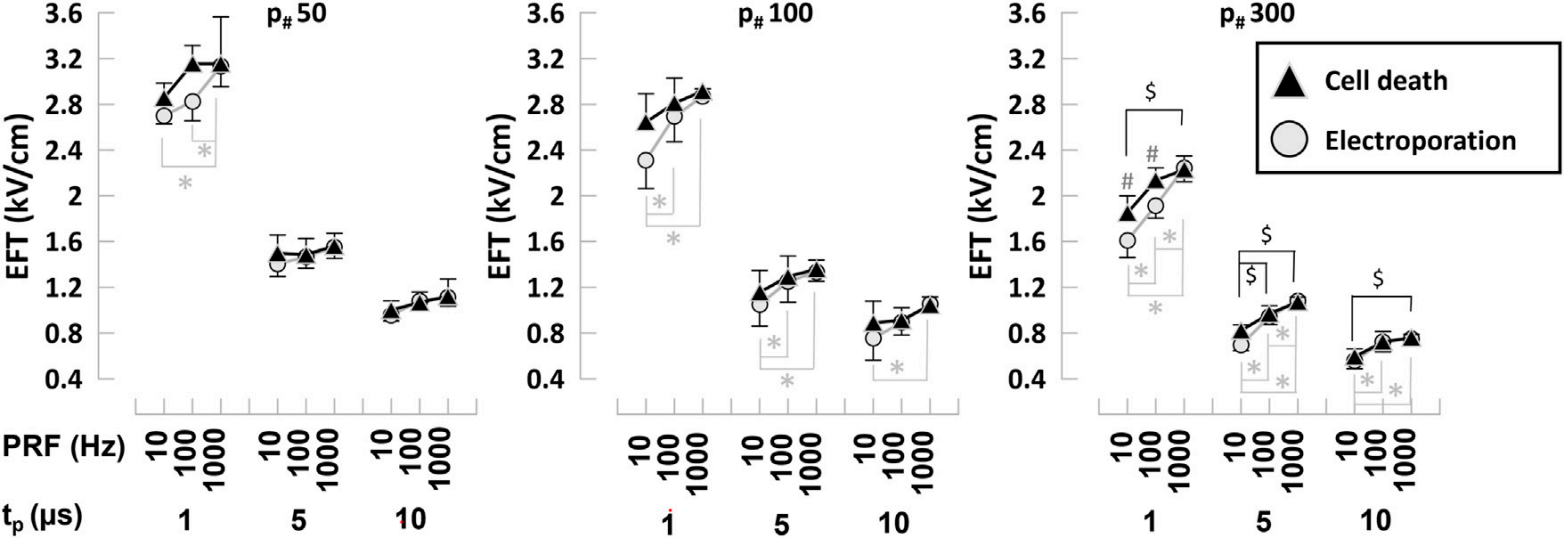
Electroporation and cell death EFTs for various PEF parameters in hiPSC-CMs. Electroporation and cell death EFTs for all the combinations of PEF parameters tested. Trains of 50, 100, and 300 pulses (left to right panels) and t_p_ = 1, 5, and 10 µs were applied at PRF = 10, 100, and 1,000 Hz to hiPSC-CMs. EFTs were calculated from electroporation and cell death areas that were quantified from the borders of YP1 and PI uptake 30 min and 4 h after PEF treatments, respectively. See text for more details. The error bars represent the standard error (95% confidence interval) for a sample size of *n* = 3–8 for all data points, *^,$,#^ *p* < .05, where * indicates the statistical analysis for electroporation, $ for cell death and # the statistical analysis comparing electroporation and cell death.

EFTs for electroporation and cell death of hiPSC-CMs were evaluated from YP1 and PI staining, respectively, for a wide range of PEF parameters. Pulses with t_p_ = 1, 5, and 10 µs were delivered in trains of *p*_#_ = 50, 100, and 300 at PRF = 10, 100, and 1,000 Hz. The EFT for cell death was inversely proportional to t_p_ and *p*_#_, and directly proportional to the PRF (Figure 4; Table 1). For example, we found that for hiPSC-CMs an increase of t_p_ from 1 to 5 µs (*p*_#_ = 50, PRF = 1,000 Hz) produced a decrease of EFT for cell death from 3.15 to 1.56 kV/cm. Increasing the *p*_#_ in the train reduced the EFT for cell death, with values down to 2.23 kV/cm for *p*_#_ 300 (t_p_ = 1 µs, PRF = 1,000 Hz). The EFT for cell death increased from 2.86 to 3.15 kV/cm when the PRF increased from 10 to 1,000 Hz (t_p_ = 1 µs, *p*_#_ = 50). The EFT for cell electroporation was consistently lower than the EFT for cell death (Figure 4).

**TABLE 1:** Summary table reporting pulsed electric field treatment endpoints for the range of PEF parameters investigated in human induced pluripotent stem cell-derived cardiomyocytes. For all combinations of PEF parameters tested, we reported the total treatment time (TT), electroporation (EP) and cell death (IRE) areas and EFTs, the EFTs ratio electroporation/cell death %, the measured maximum temperature change (ΔT_Max_) and the 50% recovery time (t_50%_), the calculated absorbed dose (AD) and adiabatic heating (AH), the treatment ranking by WSM and TOPSIS methods. For all the endpoints tabled, we reported the average and standard error.

V_p_ (V)	t_p_ (µs)	*p*_#_	PRF (Hz)	TT (s)^a^	EP area (mm^2^)	IRE area (mm^2^)	EP EFT (kV/cm)	IRE EFT (kV/cm)	EFTs ratio%	ΔT_max_ (°C)	Δt_50%_ (s)	AD (J/g)	AH (°C)	WSM	TOPSIS
568	1	50	10	5	1.8 ± .12	1.56 ± .19	2.7 ± .07	2.86 ± .13	94.48	3.1 ± .1	5.8 ± .2	18.8 ± 1.7	4.5 ± .5	26	22
			100	.5	1.62 ± .27	1.15 ± .21	2.83 ± .17	3.16 ± .16	89.56	3.6 ± .1	4.0 ± .2	22.9 ± 2.3	5.5 ± .6	17	26
			1,000	.05	1.18 ± .24	1.16 ± .52	3.13 ± .18	3.15 ± .41	99.33	3.5 ± .1	4.0 ± .1	23.1 ± 6	5.5 ± 1.5	16	20
284	5	50	10	5	1.99 ± .26	1.78 ± .35	1.4 ± .11	1.5 ± .16	93.71	3.1 ± .1	5.7 ± .2	26 ± 5.4	6.2 ± 1.3	7	12
			100	.5	1.84 ± .21	1.8 ± .31	1.47 ± .1	1.49 ± .14	98.68	3.6 ± .1	3.8 ± .2	25.6 ± 4.7	6.2 ± 1.2	5	5
			1,000	.05	1.65 ± .2	1.63 ± .22	1.55 ± .1	1.56 ± .11	99.34	3.6 ± .1	3.7 ± .1	28.2 ± 3.7	6.8 ± .9	6	8
236	10	50	10	5	2.73 ± .17	2.52 ± .25	.96 ± .05	1 ± .08	95.4	3.7 ± .5	5.5 ± .2	23.2 ± 3.7	5.6 ± .9	2	2
			100	.5	2.23 ± .18	2.28 ± .27	1.08 ± .06	1.07 ± .09	101.33	5.4 ± .7	3.4 ± .3	26.3 ± 4	6.3 ± 1	1	1
			1,000	.05	2.1 ± .21	2.14 ± .45	1.11 ± .08	1.12 ± .15	99.41	5.9 ± .2	3.1 ± .2	29.4 ± 7.3	7 ± 1.8	3	3
568	1	100	10	10	2.49 ± .26	1.91 ± .38	2.31 ± .14	2.64 ± .25	87.46	4.5 ± .1	7.1 ± .1	32.4 ± 6.4	7.8 ± 1.6	23	27
			100	1	1.82 ± .22	1.64 ± .32	2.69 ± .14	2.81 ± .22	95.85	5.6 ± .1	3.8 ± .2	36.5 ± 5.5	8.8 ± 1.4	20	23
			1,000	.1	1.54 ± .14	1.47 ± .02	2.87 ± .1	2.92 ± .02	98.43	5.7 ± .1	3.5 ± .2	39.1 ± .4	9.4 ± .1	22	21
284	5	100	10	10	3.04 ± .28	2.71 ± .61	1.05 ± .08	1.16 ± .19	90.81	6.5 ± .1	6.6 ± .1	31.4 ± 9.9	7.5 ± 2.4	10	9
			100	1	2.38 ± .21	2.27 ± .47	1.25 ± .08	1.29 ± .18	96.67	8.1 ± .2	3.5 ± .2	38.8 ± 10.9	9.3 ± 2.7	8	6
			1,000	.1	2.15 ± .16	2.09 ± .19	1.33 ± .06	1.36 ± .08	98.19	7.6 ± .1	3.4 ± .2	42.5 ± 4.5	10.2 ± 1.1	12	10
236	10	100	10	10	3.68 ± .52	3.01 ± .82	.75 ± .08	.89 ± .19	84.71	6.9 ± .1	6.9 ± .3	37.1 ± 14.9	8.9 ± 3.6	9	11
			100	1	2.96 ± .35	2.88 ± .42	.89 ± .09	.91 ± .11	97.98	9.5 ± .1	3.2 ± .2	38.6 ± 9.5	9.3 ± 2.3	4	4
			1,000	.1	2.38 ± .52	2.36 ± .24	1.05 ± .14	1.05 ± .07	100.87	9.3 ± .4	3.2 ± .3	50.5 ± 6.2	12.1 ± 1.5	11	7
472	1	300	10	30	3.39 ± .27	2.77 ± .33	1.61 ± .1	1.85 ± .15	86.99	5.3 ± .1	10.9 ± .2	47.5 ± 7.5	11.4 ± 1.8	27	24
			100	3	2.61 ± .21	2.15 ± .21	1.91 ± .09	2.13 ± .11	89.69	10.2 ± .2	4.3 ± .1	63 ± 6	15.1 ± 1.5	24	25
			1,000	.3	1.95 ± .24	1.98 ± .22	2.24 ± .14	2.23 ± .12	100.65	8.8 ± .3	3.6 ± .2	68.7 ± 7.3	16.5 ± 1.8	25	19
236	5	300	10	30	4.03 ± .4	2.68 ± .34	.7 ± .07	.82 ± .05	84.59	5.5 ± .3	12.5 ± .7	46.8 ± 5	11.2 ± 1.2	21	16
			100	3	2.73 ± .17	2.64 ± .25	.94 ± .04	.97 ± .07	97.35	11.1 ± .2	3.7 ± .1	65.2 ± 9.3	15.6 ± 2.3	13	14
			1,000	.3	2.24 ± .12	2.26 ± .13	1.08 ± .04	1.07 ± .04	100.51	10.0 ± .2	2.6 ± .1	79.6 ± 5.7	19.1 ± 1.4	18	18
144	10	300	10	30	3.09 ± .23	2.88 ± .43	.56 ± .04	.59 ± .07	94.4	4.3 ± .1	12.8 ± .2	48.9 ± 10.9	11.7 ± 2.7	19	13
			100	3	2.1 ± .34	2.11 ± .46	.73 ± .07	.72 ± .09	100.35	9.8 ± 1.1	4.2 ± .2	73.1 ± 17.4	17.6 ± 4.2	14	15
			1,000	.3	1.95 ± .3	1.9 ± .12	.75 ± .06	.76 ± .03	99.15	9.4 ± .1	3.2 ± .6	79.3 ± 4.7	19 ± 1.2	15	17

^a^ TT = total treatment time

To assess the proportion of electroporated and dead cells, we computed the ratio in percent of the EFTs determined by YP1 and PI staining. The EFT ratio was between approximately 80%–100%, showing a larger population of dead cells with increasing PRF and t_p_ (Table 1). As this ratio approaches 100%, the electroporation and cell death areas reach a comparable surface, ideal for ablation treatments where reversible effects are not desired.

Reduction in the EFT for cell death and in reversible electroporation beyond the cell death region are only two of the criteria to be considered when designing ablation strategies. PEF can induce heating that should be minimized to avoid cell death by thermal damage. Since temperature increase due to Joule heating is proportional to the absorbed doses, energy delivery minimization should be considered when selecting PEF treatment parameters.

**FIGURE 5:**
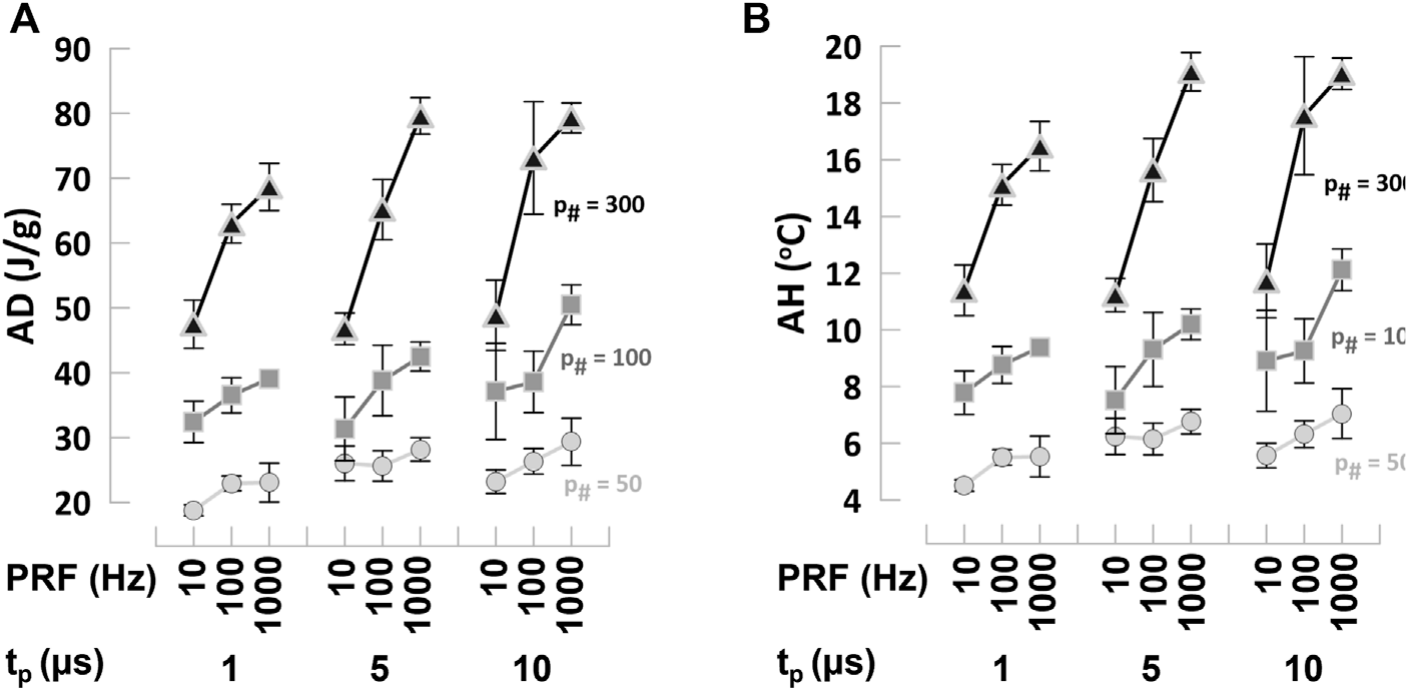
Absorbed dose and adiabatic heating at the EFT for cell death in hiPSC-CMs **(A)** Absorbed dose (AD) and **(B)** adiabatic heating (AH) calculated from cell death EFTs for all the combinations of PEF parameters tested. Trains of 50, 100, and 300 pulses (left to right panels) and t_p_ = 1, 5, and 10 µs were applied at PRF = 10, 100, and 1,000 Hz to hiPSC-CMs. See text for more details. Symbols legend as labeled: *p*_#_ = 50 light gray circles, *p*_#_ = 100 gray squares, *p*_#_ = 300 black triangles. The error bars represent the standard error for a sample size of *n* = 3–8 for all data points.

To evaluate the energy deposition by combinations of PEF parameters, we calculated the AD at the cell death EFT as described in (Ibey et al., 2009). Variation in PEF parameters produce significant changes in the AD that ranged from approximately 20–80 J/g (Table 1). For all PEF combinations, trains with lower *p*_#_ achieved cell killing at lower doses, i.e., 18.8–29.4 J/g for 50 *p*_#_ compared to 46.8–79.9 J/g for 300 *p*_#_; for the same *p*# in the train, the reduction in t_p_ and PRF decreased the delivered dose due to the lower energy deposition by shorter and less frequent pulse applications (Figure 5A). High ADs might result in a non-negligible temperature increase that should be considered a factor when planning PEF treatments.

These results demonstrated that PEF parameters can be modulated to reduce the EFT for cell death, undesired reversible effects, as well as the energy deposition during treatment. However, the high values of AD reached for certain pulsing conditions indicated that temperature assessment is suggested during PEF ablation.

### 3.3 Temperature changes during PEF treatments

PEF-induced temperature changes (Figure 1B) were measured in proximity to one of the electrodes (Supplementary Figure S2). PEF treatments induced a ΔT_max_ that ranged between 3°C and 11°C (corresponding to an absolute increase in temperature from 37°C baseline to 40°C and 49°C) and a Δt_50%_ that ranged between 3.2 and 12.8 s. The highest ΔT_max_ measured was 11°C and the maximum Δt_50%_ 12.8 s when a train of *p*_#_ = 300, PRF = 100 Hz, t_p_ = 5 µs, and V_p_ = 236 V was applied. For the longest train tested, i.e., *p*_#_ = 300 at PRF = 10 Hz, the recovery time was less than 30 s (Table 1). In general, ΔT_max_ increased with *p*_#_, and t_p_, while PRF showed a variable trend with often a peak at 100 Hz, suggesting that the temperature probe, due to its response time, may underestimate the highest temperature increase for high repetition rates, e.g., f = 1,000 Hz. To estimate the maximum temperature increase possible independently of specific environmental and experimental factors, we also computed the AH at the cell death EFT, as described in (Ibey et al., 2009). For low 50 and 100 *p*_#_ the AH was moderate and ranged between 4°C and 12°C (Figure 5B; Table 1). However, for higher PEF doses, temperature values reached 20°C, likely resulting in some thermal damage (Davalos et al., 2005).

### 3.4 Selection of PEF parameters for ablation of HiPSC-CMs

Our results demonstrated that this assay allows for the quantification of critical factors in the selection of PEF parameters for cardiac ablation treatments. In order to select the optimal parameters for PEF cardiac ablation among the combinations studied in this work, we ranked the PEF treatments with two multiple-criteria decision analysis methods. Both the Weight Sum Model (WSM; also known as weighted linear combination or simple additive weighting) and Technique for Order of Preference by Similarity to Ideal Solution (TOPSIS) are multi-criteria decision analysis methods used to rank the 27 various scenarios or “alternatives” consisting of multiple metrics or “criteria”. The WSM method uses a weighted sum to determine ranking, while TOPSIS uses L2-distance to measure distance to a best-case scenario of the reported criteria (Fishburn, 1967; Tzeng and Huang, 2011). Before processing our data with WSM and TOPSIS, weights to each of the critical factors considered were assigned arbitrarily considering relevance to clinical applications: 5 to the EFT for cell death, 3 to AH, 2 to AD, 2 to ratio electroporation/cell death EFTs, 1 to the total treatment time. Our analysis using both methods ranked the following treatments as the top two: i) *p*_#_ = 50, t_p_ = 10 µs, PRF = 100 Hz, and ii) *p*_#_ = 50, t_p_ = 10 µs, PRF = 10 Hz. These PEF treatments were characterized by an EFT for cell death equal to 1.07 and 1.00 kV/cm, an AH of 6.3°C and 5.6°C, an AD of 26.3 and 23.2 J/g, respectively, a ratio electroporation/cell death of 100% and 95%, and a total treatment time of .5 and 5 s (Table 1).

## 4 Discussion

### 4.1 Feasibility of PEF treatments in HiPSC-CMs

In this study, we established a robust high-throughput experimental protocol for efficient testing of a wide range of PEF parameters in a commercially available *in vitro* human cardiomyocyte model for cardiac ablation treatment optimization. PEF-based cardiac ablation devices have been gaining increasing interest for the treatment of AF in patients that do not respond to pharmaceuticals. However, the lack of preclinical standardized methods to optimize PEF parameter selection for safe and efficient patient therapy has slowed device development and regulatory decision-making. Previous studies relied primarily on animal models or various cell lines and reported a limited range of PEF parameters (Xie et al., 2015; Xie and Zemlin, 2016; Semenov et al., 2018; Azarov et al., 2019; Neuber et al., 2019; Heller et al., 2021; Baena-Montes et al., 2022). Here, we report on an assay that enables the quantification of electroporation and cell death in hiPSC-CM monolayers following the application of PEF treatments with varying pulse parameters. Combined with temperature measurements, this approach can facilitate optimal PEF parameter selection ensuring complete PEF-induced cardiac ablation while minimizing undesired secondary effects (e.g., reversible effects and thermal damage).

### 4.2 Considerations for parameters selection in PEF treatments

Cell electroporation and cell death by PEF are threshold phenomena for which reversible and irreversible cell permeabilization occur only if the applied electric field in a tissue reaches a certain value. While increasing V_p_ is the most direct way to increase the ablation volume, it also increases the chances of muscle contraction and temperature effects. Minimization of the EFT for cell death by tuning different pulse parameters enables achievement of the desired ablation volume while keeping the V_p_ low (Sano et al., 2018). Additionally, for ablation procedures, it is desirable to select PEF parameters able to reduce the volume of reversible electroporation that could cause stimulation outside the ablation-targeted region or stunning and consequent recurrence of AF. Finally, this parameter selection should guarantee that the temperature of the tissue remains at or below physiological ranges to avoid thermal damage.

In previous studies, needle electrodes producing a non-uniform electric field distribution have been used to identify the EFT of electroporation and cell death in cell monolayers and in 3D *in vitro* models (Arena et al., 2012; Sano et al., 2018; Aycock et al., 2022; Gudvangen et al., 2022). These studies demonstrated that the EFTs can be quantified by matching the area of the stained surface obtained experimentally to the electric field map obtained numerically. Using this approach, we quantified the electroporation and cell death EFTs of hiPSC-CMs for biphasic pulses with t_p_ = 1, 5, and 10 µs delivered in trains of *p*_#_ = 50, 100, and 300 at PRF = 10, 100, and 1,000 Hz. Consistent with a previous study on a different cell type (Gudvangen et al., 2022), we found that for hiPSC-CMs t_p_ was the PEF parameter that mostly influenced the EFT for both electroporation and cell death with an inversely proportional relationship. As the t_p_ increased by one order of magnitude, the EFTs decreased non-linearly approximately 3 fold (see Table 1 for absolute values). Conversely, increasing the number of pulses in the train from 50 to 300 produced a decrease in the electroporation and cell death EFTs of only 1.4–1.8 fold (see Table 1 for absolute values). Preliminary data extending our results to *p*_#_ = 500 resulted in plateauing of the EFTs (data not shown), suggesting that adding pulses to the train only produced additional heating without further widening of the electroporation and cell death areas, i.e., lowering of the EFTs. In the range of the pulse parameters selected, increase of the PRF by three orders of magnitude led to a moderate but significant increase of the EFTs.

When designing a PEF-based ablation treatment, the EFT for electroporation compared to cell death indicates possible transitory effects, such as action potential (AP) generation, or stunning near the ablation area. In our study, the minimum ratio of EFTs for electroporation and cell death was 84%, showing generally a larger population of dead cells than of reversibly electroporated cells.

Another consideration during the implementation of PEF ablation treatments is the temperature increase due to Joule heating. While it is often suggested that IRE ablation is a thermal damage-free approach, PEF can produce thermal effects if treatment parameters are not selected correctly. We monitored temperature changes throughout the experiments and measured a maximum temperature increase of 11°C and maximum recovery time of 13 s. However, these measurements are dependent on the specific experimental environment. To estimate the worst-case scenario for temperature increase, we computed the AH at the cell death EFT. AH calculations, in fact, provide the maximum possible temperature increase, since heat dissipation and heat losses are not allowed. For low pulse numbers in the train, the AH ranged between 4°C and 12°C, remaining below the threshold for instant cell death by thermal damage (Davalos et al., 2005). While the quantitative assessment of the actual thermal damage to PEF-exposed cells was outside the scope of this paper, for higher doses the AH reached 20°C, indicating that some degree of cell death by thermal damage, especially close to the electrodes where the electric field is higher, cannot be ruled out (Davalos et al., 2005; Garcia et al., 2011; Long et al., 2014).

In summary, our results demonstrated that this assay allows for the quantification of critical factors for the selection of PEF parameters for cardiac ablation treatments, such as the EFT for cell death, the ratio of EFT for electroporation/cell death, and the temperature increase due to PEF treatment. For example, a train of 300 pulses with t_p_ = 10 µs, PRF = 10 Hz, with a total treatment time of 30 s, resulted in the lowest EFT for cell death measured (.59 kV/cm), with a ratio for EFT of electroporation/cell death at 94.4%; however, the AD was significant (48.9 J/g) as was the temperature increase (4.3°C measured, 11.7°C calculated).

In fact, minimization of the EFT for cell death should not be the only indicator for the selection of PEF treatments. One possible criterion is the identification of the set of parameters that minimizes the EFT for cell death and maintains the ratio of electroporation/cell death close to 100%, while reducing the AD, the thermal increase, and the total treatment time. One way to do this is to rank PEF treatments with a multiple-criteria decision analysis. Our analysis, using both WSM and TOPSIS methods, indicted that the top two PEF parameter combinations that best matched the selection criterion above were *p*_#_ = 50, t_p_ = 10 µs, PRF = 10, and 100 Hz. These two combinations, with respect to the one selected only based on the minimum cell death EFT, showed a drastically reduced treatment time, (i.e., 6-, 60-fold lower), up to 5% higher ratio of electroporation/cell death EFTs, and an almost 50% reduction in temperature increase and AD, with only a modest 1.8-fold increase in EFT for cell death (see Table 1 for absolute values).

### 4.3 Study limitations and future work

One limitation of our study is the relatively restricted range of PRF values investigated, due to the capabilities of our pulse generator. Extending our results to higher PRF values could cover common treatments in preclinical studies, such as H-FIRE, i.e., high-frequency IRE, approaches that deliver tens of pulses in the kHz–MHz range (Arena et al., 2011), and cutting-edge nanosecond PEF approaches (Gudvangen et al., 2022).

Cell detachment due to PEF application is often observed during electroporation experiments, especially in excitable cells with contractile properties. PEF can elicit APs by either direct effects on voltage gated channels or as a downstream effect of cell electroporation that leads to loss of resting membrane potential (Casciola et al., 2019). In cardiac cells, this ionic imbalance can lead to contraction and eventual detachment from the plate. To enhance cell adherence, and to avoid cell detachment and peeling of the monolayer, we used patterned nanofiber plates. We cannot rule out some degree of undesired membrane damage during cell contraction induced by PEF application, which occurred due to the limited elasticity of the plates used (Graybill et al., 2021). This could be one possible explanation for the unexpected gradual PI uptake we observed in our experiments. To mitigate this limitation, future work will investigate the impact of exposing hiPSC-CM monolayers cultured on more flexible substrates, such as PDMS (Herron et al., 2016), on PEF treatments.

While PFA has been mostly proposed for the treatment of AF, our human model is composed of multiple cardiac cell subtypes, (i.e., ventricular, atrial, nodal). Approximately 50%–80% of the population is ventricular and the remaining 20%–50% is a combination of nodal and atrial (Ma et al., 2011). To the best of our knowledge, limited information is available on different sensitivities of atrial and ventricular cardiomyocytes to PEF, with some studies suggesting that atria are more susceptible to electroporation than ventricles (Fedorov et al., 2008; Neven et al., 2014). Currently, it is not clear if this difference results from their decreased thickness and increased heterogeneity (macroscopic tissue properties), or from different membrane shape and structure (cellular properties). While this could be relevant in the assessment of tissue specificity to PEF, we would not expect significant changes in the general trends reported here.

Finally, our assay is based on a 2D *in vitro* model, while clinical applications involve 3D *in vivo* tissues. The quantitative results presented here might differ for *in vivo* treatments, but general trends regarding how the thresholds change with pulse parameters would likely be maintained (Gudvangen et al., 2022). Additional studies may be able to predict the ablated volume and corresponding EFTs for specific PEF treatments in 3D tissues based on the results from our 2D *in vitro* assay. To support translatability of the results obtained with this *in vitro* assay to *in vivo* models, we are currently implementing a computational, i.e., *in silico*, cardiac model that will be coupled with the EFTs identified in this work to predict specific ablation areas for combinations of PEF parameters. The *in silico* results will be validated with experiments on isolated perfused porcine hearts by histological assessment of ablated tissues, justifying the *in vitro* assay as a reliable surrogate for *in vivo* testing.

## 5 Conclusion

This work paves the way for a standard preclinical assay supporting early non-clinical development of PEF-based cardiac ablation devices and informing regulatory decision-making. Here, we demonstrated several important findings, including: 1) hiPSC-CMs respond to PEF treatments showing both acute (electroporation) and long-term (cell death) effects; 2) variations in PEF parameters are reflected in quantifiable variations of electroporation area size and EFTs; 3) optimal PEF parameters can be selected by combining results from EFTs and the assessment of temperature increase and reversible effects. Furthermore, this assay can be translated to different cell types, including tissues anatomically adjacent to the heart, to investigate tissue specificity to PEF ablation.

## Data Availability

The original contributions presented in the study are included in the article/Supplementary Material, further inquiries can be directed to the corresponding author.
