# Supplementary material for: Human in vitro assay for irreversible electroporation cardiac ablation
Source: Front Physiol. 2023 Jan 9;13:1064168. doi: 10.3389/fphys.2022.1064168 (PMC9869257; doi:10.3389/fphys.2022.1064168)
Supplement: Supplementary file 1 [file DataSheet1.docx]

Supplementary Material

# Supplementary Figures

## Assement of confluency and integrity of hiPSC-CM monolayers before PEF treatments


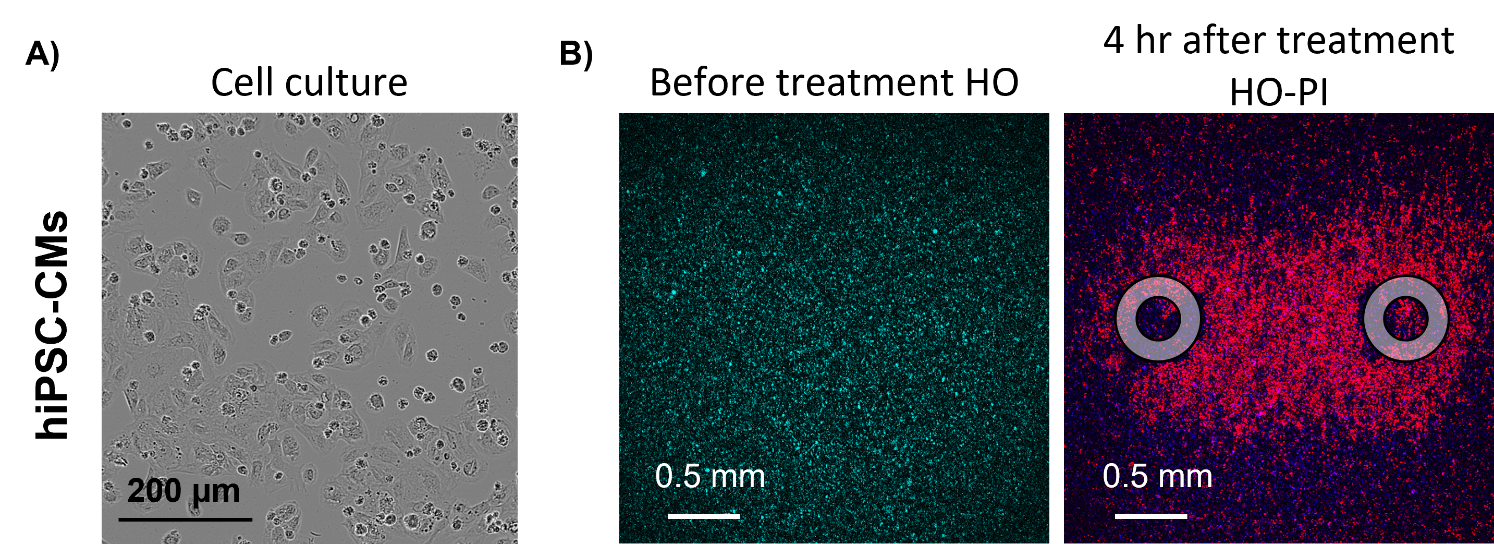


**Supplementary Figure 1.** A) Representative bright field image of hiPSC-CMs plated on plastic bottom 96-well plates at a concentration of ~55k cells per well following manufacturer’s instructions and adjusted to 110-115k cells per well to account for increased surface in 96-well Nanofiber plates. B) Representative, fluorescence images obtained with confocal microscopy show high confluency and integrity of the hiPSC-CM monolayer before treatment: (Left) Cell nuclei were stained with Hoechst-33342 dye (Ho, blue) and imaged before PEF treatment; (Right) irreversibly electroporated cells were stained with Propidium Iodide (PI, red) and imaged 4 hr after treatment. Gray circles indicate the footprints of the electrodes positioned orthogonally to the cell monolayer.

## High-throughput experimental setup for electroporation of cell monolayers and temperature measurements


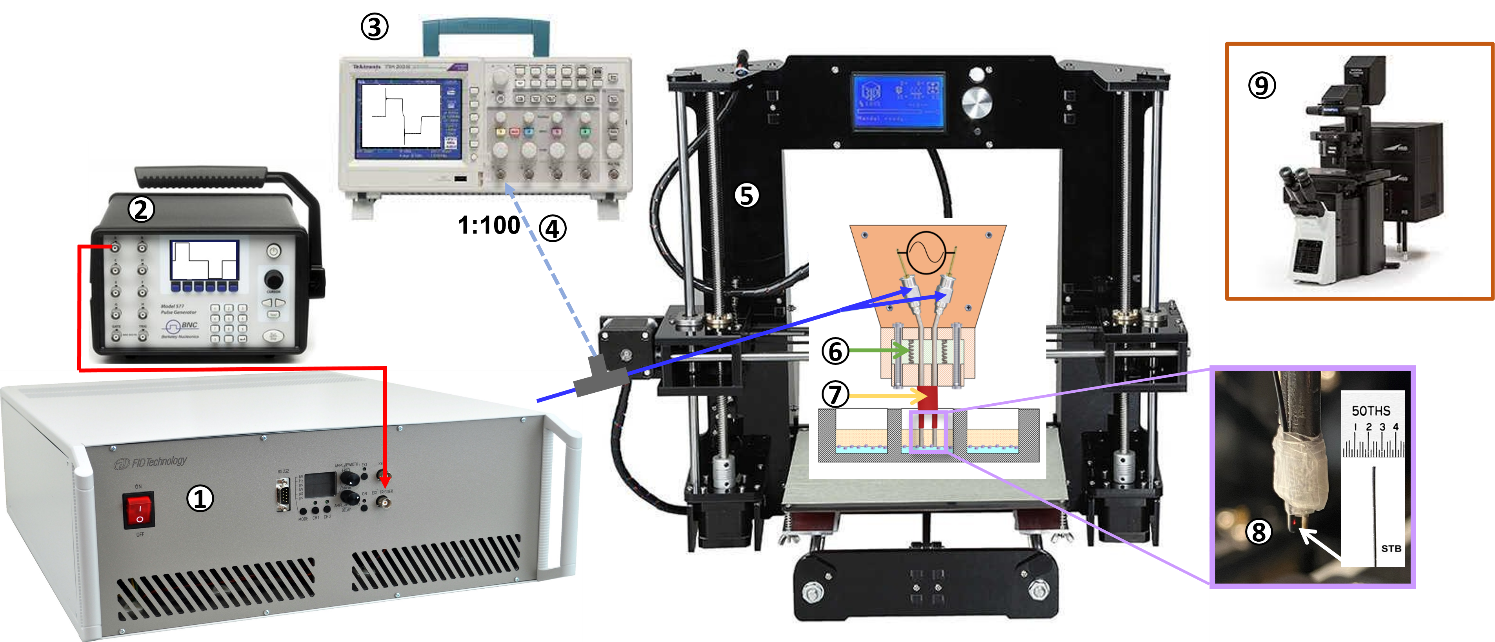


**Supplementary Figure 2.** A custom PEF voltage generator (1) was triggered by a digital delay generator (2) to produce the desired PEF treatment. An oscilloscope (3) with attenuation probe 1:100 (4) was used to measure the waveform applied at the cell monolayer. A 3D printer (5) was utilized as a robotic arm and equipped with a spring system (6) to minimize the contact of the electrodes (7) with the bottom of the multi well plate. The temperature increase due to PEF treatments was monitored using an optic probe (8) placed between the electrodes, parallel and adjacent to one of them. A confocal microscope (9) was used to acquire images 30 minutes and 4 hours after PEF treatments.

## Phase voltage selection for different combination of PEF parameters


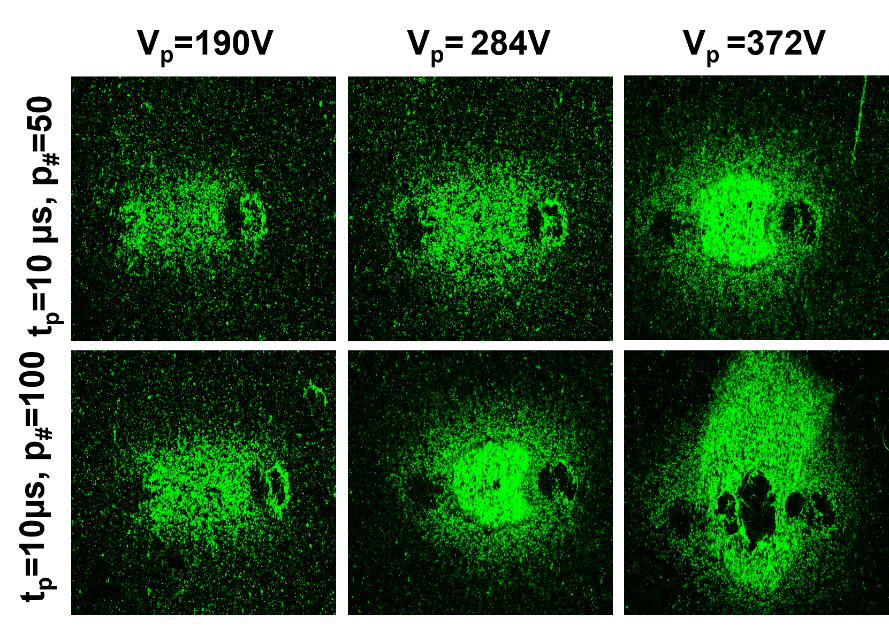


**Supplementary Figure 3.** Representative fluorescent images (YP1 staining) are shown for t_p_ 10 µs, PRF 1000 Hz, p_#_ 50 (top images) and 100 (bottom images). V_p_ was varied to target a detectable effect while avoiding arcing at the electrodes and detachment in hiPSC-CMs.

## Determination of cell death (i.e., IRE) areas by red pixel count


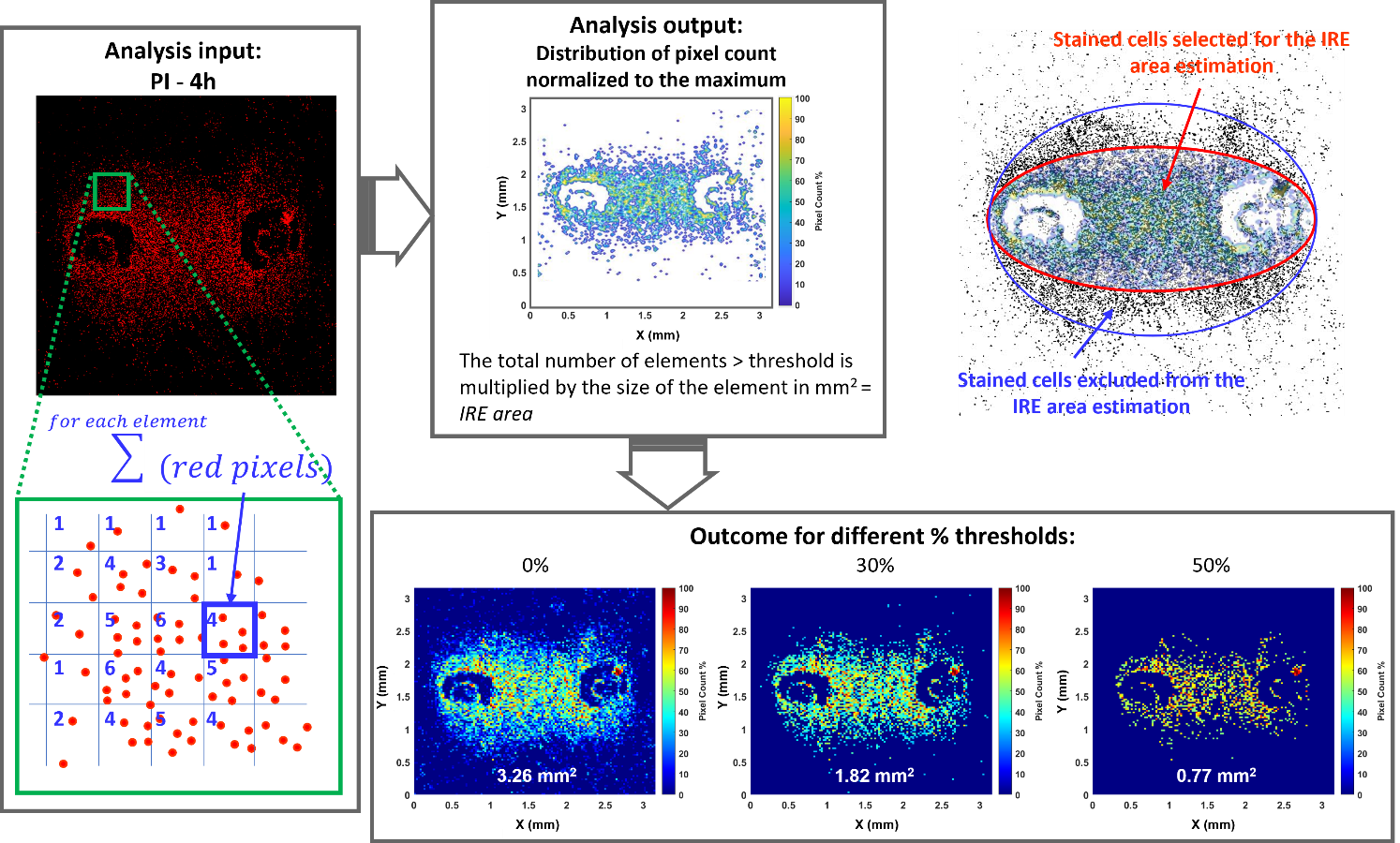


**Supplementary Figure 4.** Images were discretized with a grid of 128 x 128 elements. For each element of the grid, the sum of pixels with intensity above background was computed and the pixel count was normalized to maximum. The sum of the total number of grid elements with pixel count above a given threshold was computed and multiplied by the dimension in mm^2^ of a single grid element obtaining the cell death (e.g., IRE) area. The bottom panel shows representative outcomes of the code for 0%, 30%, and 50% thresholds (Supplementary Appendix 1).

## Electric field distribution produced by needle electrodes at the plane of the hiPSC-CMs monolayer


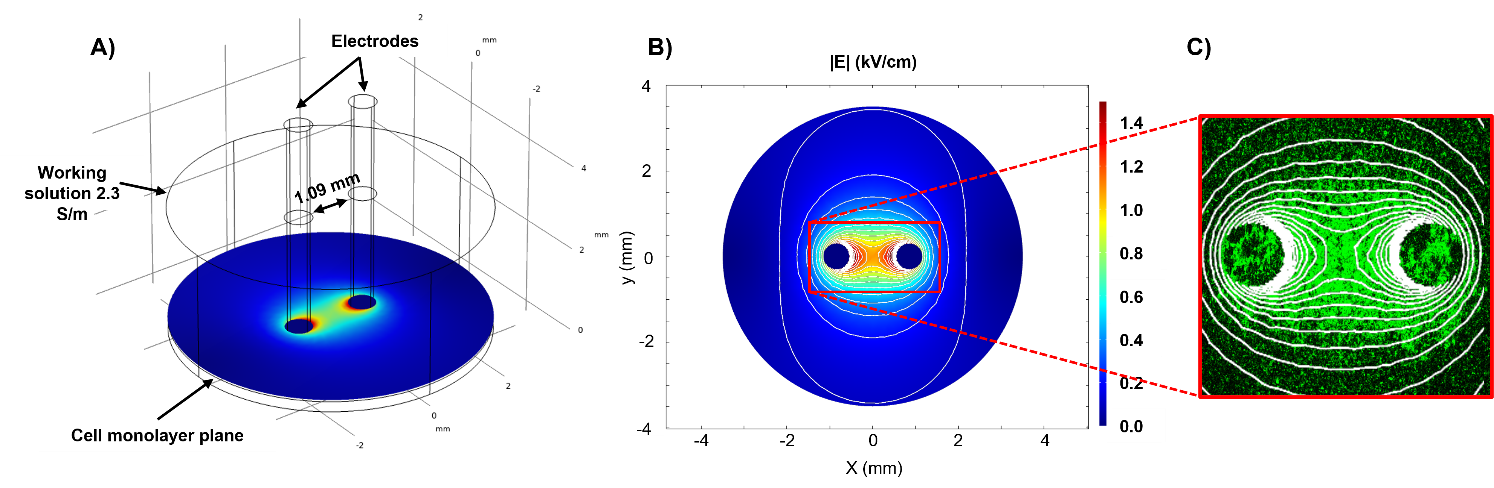


**Supplementary Figure 5.** A) Geometry and materials of the modeled experimental setup. 0.61 mm diameter electrodes were placed orthogonally to the bottom of the well, with an edge-to-edge distance of 1.09 mm and inserted in a cylinder with electric conductivity 2.3 S/m, to mimic experimental conditions. B) Representative non uniform electric field distribution at the cell monolayer plane when 144V were applied to the electrodes. Isolines are shown as white solid lines. C) Example of electric field isolines from the electric field intensity distribution maps and a fluorescent image compared to determine the EFT for a given PEF treatment.

# Supplementary Tables

## PI stained area measured at different time intervals after PEF treatment to determine the timepoint for cell death in hiPSC-CMs

| **Time, hr** | 0.5 | 2 | 4 | 6 | 24 |
| --- | --- | --- | --- | --- | --- |
| **Dead cells area, mm^2^** | 0.92±0.02 | 1.57±0.04 | 1.53±0.05 | 1.49±0.06 | 1.53±0.05 |

**Supplementary Table 1:** HiPSC-CMs were exposed to a train of 100 pulses, V_p_ = 160V, t_p_ = 3µs, p_#_ 100, PRF = 1kHz. Monolayers were stained with PI at different timepoints after PEF treatment to assess the cell death region over time. Data are mean ± SEM for a sample size of n = 3.

# Appendix 1

Annotated MATLAB code for PI image analysis is reported below.

rgbImage = imread(image.tif'); %Open the fluorescence image

imshow(rgbImage) %Display the fluorescence image

redChannel = rgbImage(:, :, 1); %Extract the red channel

s=length(redChannel); %Determine the size of the image

ds=4; %Define discretization step

n=s/ds; %Define the size of the matrix

th=x; %Pixel intensity threshold for background subtraction – substitute to x a value from 0 to the maximum level of pixel intensity

th1=y; %Density count threshold – substitute to y a value from 0 to 100

cf= 3181.981/n*10^-3; %conversion factor px to mm

cfa= 3181.981*3181.981/(n*n)*10^-6; %conversion factor area px to mm^2

dc = zeros(n); %Initialization of density_count matrix

%Add a count (+1) to the each element of the density_count matrix if the intensity of the red pixel is above th

for i=0:n-1

for ii=0:n-1

for j=i*ds+1:(i+1)*ds

for k=ii*ds+1:(ii+1)*ds

if greenChannel(j,k)>=th dc(i+1,ii+1)=dc(i+1,ii+1)+1;

end

end

end

end

end

M=max(dc(:)); %Find the density_count maximum

DC=dc/M*100; %Normalize the density_count matrix to maximum

%Sum of the pixels in the matrix - exclude the elements where the count is below th1

DCth=DC;

DCth(DCth < th1) = 0;

Pixel_count_DCth = sum(DCth~=0,2);

Pixel_count_total_DCth = sum(Pixel_count_DCth);

DCth_area=cfa*Pixel_count_total_DCth %Convert the sum of the pixels from px to mm^2

x_axis = [0:n-1]*cf; %Convert the axis x from px to mm

y_axis = [0:n-1]*cf; %Convert the axis y from px to mm

%Plot density_count matrix after background subtraction

figure

surf(x_axis,y_axis,DC,'EdgeColor','none');

view(0,90)

xlabel('x (mm^2)','rotation',0)

ylabel('y (mm^2)','rotation',90)

xlim([0 x_axis(end)])

ylim([0 y_axis(end)])

grid off

c = jet;

colormap(c);

cb = colorbar;

cb.Label.String = 'Pixel Count %';

set(gca,'FontSize',12,'fontweight','bold')

%Plot density_count matrix after threshold application

figure

surf(x_axis,y_axis,DCth,'EdgeColor','none');

view(0,90)

xlabel('x (mm^2)','rotation',0)

ylabel('y (mm^2)','rotation',90)

xlim([0 x_axis(end)])

ylim([0 y_axis(end)])

grid off

c = jet;

colormap(c);

cb = colorbar;

cb.Label.String = 'Pixel Count %';

set(gca,'FontSize',12,'fontweight','bold')

disp('End')
